# Comparison of the ‘Chemical’ and ‘Structural’ Approaches to the Optimization of the Thrombin-Binding Aptamer

**DOI:** 10.1371/journal.pone.0089383

**Published:** 2014-02-20

**Authors:** Olga Tatarinova, Vladimir Tsvetkov, Dmitry Basmanov, Nikolay Barinov, Igor Smirnov, Edward Timofeev, Dmitry Kaluzhny, Andrey Chuvilin, Dmitry Klinov, Anna Varizhuk, Galina Pozmogova

**Affiliations:** 1 Institute for Physical-Chemical Medicine, Moscow, Russia; 2 Orekhovich Institute of Biomedical Chemistry, Moscow, Russia; 3 Topchiev Institute of Petrochemical Synthesis, Moscow, Russia; 4 Engelhardt Institute of Molecular Biology, Moscow, Russia; 5 Shemyakin-Ovchinnikov Institute of Bioorganic Chemistry, Moscow, Russia; University of Houston, United States of America

## Abstract

Noncanonically structured DNA aptamers to thrombin were examined. Two different approaches were used to improve stability, binding affinity and biological activity of a known thrombin-binding aptamer. These approaches are chemical modification and the addition of a duplex module to the aptamer core structure. Several chemically modified aptamers and the duplex-bearing ones were all studied under the same conditions by a set of widely known and some relatively new methods. A number of the thrombin-binding aptamer analogs have demonstrated improved characteristics. Most importantly, the study allowed us to compare directly the two approaches to aptamer optimization and to analyze their relative advantages and disadvantages as well as their potential in drug design and fundamental studies.

## Introduction

Nucleic acid aptamers have demonstrated impressive potential as tools for molecular biology and medicinal chemistry. A large number of DNA and RNA aptamers to various kinds of targets have been reported to date, and new aptamers are continually being discovered through an in vitro selection process called SELEX (systemic evolution of ligands by exponential enrichment) [1]. Along with the design of new aptamers, much effort is devoted to the modification of known aptamers. The modification aims to overcome potential drawbacks, primarily insufficient stability, or to improve affinity and selectivity of nucleic acid aptamers. In this paper, we compare two general types of modification: chemical modification and the addition of a duplex module to the core structure. The effects of these modifications are evaluated using the model nucleic acid ligand – thrombin binding aptamer TBA15 (GGTTGGTGTGGTTGG) [2]. TBA is probably one of the best known DNA aptamers. Its spatial organization and interaction with thrombin are well characterized [3], [4], [5] and it has been employed as a model structure in a number of drug design and diagnostic design studies [6], [7]. The important advantage of the thrombin-TBA pair as a model is the relative ease of assessing their binding efficiency in biological media. TBA inhibits thrombin function upon binding, which results in decreased blood clotting time, which can be detected by a simple in vitro test (‘thrombin time test’ [8]).

## Results

### Chemical modification of the aptamer

**Figure 1:**
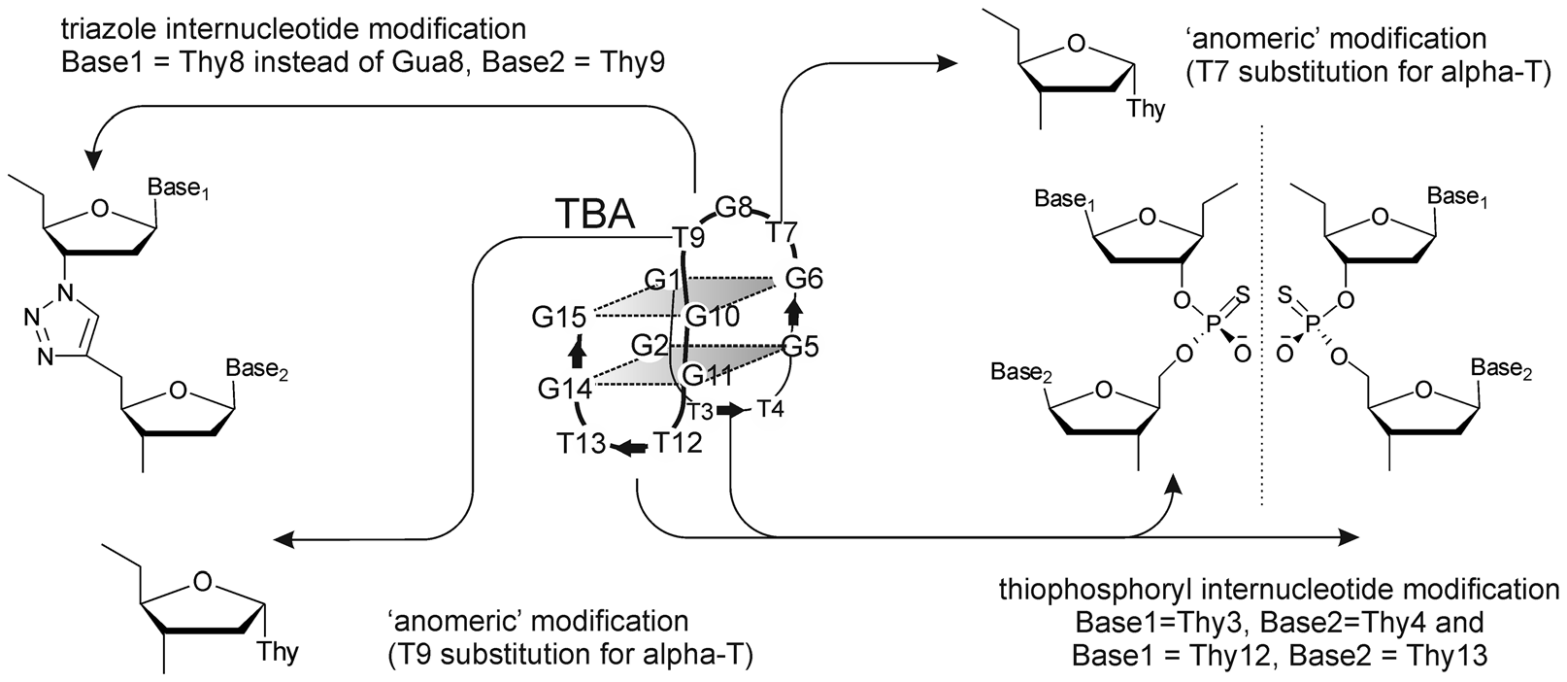
Schematic representation of the thrombin-binding aptamer and its chemical modifications.

Chemical modification was the first – and is arguably most popular – approach that we considered for TBA optimization [9]. A significant number of chemically modified TBA analogs have been reported in the last decade [10], [11], [12], [13], [14], [15]. The relative benefits of those modifications are difficult to determine based on published data because very few modified aptamers have been comprehensively investigated. We assumed that direct comparative assessment of a series of aptamers by a unified set of methods was needed for a balanced view regarding the advantages and disadvantages of chemical modifications. Some generalizations can be made, however, based on the data in the literature. In particular, analysis of the literature revealed preferred modification positions based on the aptamer 3D structure. Like many target-specific nucleic acid ligands, TBA adopts a noncanonical conformation in solution. In the presence of sodium, potassium or ammonium ions, it folds into an antiparallel two-tetrad G-quadruplex (GQ) (Figure 1) [4]. It has been shown that GQ formation is crucial for TBA binding with thrombin [10], [11], so modifications that decrease GQ thermostability (i.e. almost any substantial modification in the quadruplex core [11], [12], [13]) are undesirable. Loop modifications tend to have insignificant effects on quadruplex thermostability, but often impart increased nuclease resistance to the aptamer [11], [12], [13]. Unmodified loops are quickly degraded in blood, like all single stranded ON fragments. The GQ core is less prone to enzymatic cleavage and its chemical modification is not required. For this reason, we herein focused mainly on loop-modified aptamers.

In this study, we synthesized and compared three TBA analogs with different loop modifications (Figure 1, Table 1): the thiophosphoryl TBA analog (thio-TBA), the triazole-linked analog (triazole-TBA) and the analog bearing alpha-thymidine (alpha-TBA). Internucleotide modifications, including the thiophosphoryl modification and the triazole modification, are well known to protect oligonucleotides (ONs) from nuclease hydrolysis [11], [12], [16], [17]. The introduction of anomeric nucleoside moieties (alpha-nucleosides) has also been shown to impart increased enzymatic stability to ONs [18]. Apart from the three analogs with loop modifications we synthesized a fully-modified thio-TBA analog (f-thio-TBA).

**Table 1:** Sequences, MALDI-TOF MS data, GQ melting temperatures and thrombin-time values of chemically modified TBA analogs.

aptamer	sequence, 5’–3’	m/z, found (calculated for [M + H]^+^)	Tm at 295 nm,°C	thrombin time, s
TBA	GGTTGGTGTGGTTGG	4728 (4727)	52±1	41±2
thio-TBA	GGT_thio_TGGTGTGGT_thio_TGG	4758 (4759)	55±1	28±1
triazole-TBA**	GGTTGGTT_triazole_TGGTTGG	4674 (4674)	42±1	31±1
alpha-TBA	GGTTGG_alpha-_TG _alpha-_TGGTTGG	4725 (4727)	42±1	25±1
f-thio-TBA	G_thio_G_thio_T_thio_T_thio_G_thio_G_thio_T_thio_G_thio_ T_thio_G_thio_G_thio_T_thio_T_thio_G_thio_G	4952(4951)	45±1	11±1*

* the same as without any ON.

**The triazole-TBA sequence is different from that of TBA (the central loop is TTT instead of TGT) because the modified fragment could only be introduced using the dithymidine triazole-containing phosphoramidite block. The slightly decreased biological activity of triazole-TBA can thus be partially attributed to the change of the sequence.

GQ folding of all aptamers was confirmed by UV-melting at 295 nm. Thermal denaturation curves (Figure S1) allowed us to determine melting temperatures (Tm) of the GQs (Table 1). As evident from Table 1, all the TBA analogs except for thio-TBA were slightly less thermostable than unmodified TBA. The bioactivity of the thrombin-binding aptamers was evaluated using thrombin-time tests (Table 1). Thio-TBA, triazole-TBA and alpha-TBA appeared to be rather efficient anticoagulants, though their effects on blood clotting time (TT values) were lower than that of TBA. F-thio-TBA failed to inhibit blood coagulation. Interestingly, at a 10-fold higher concentration, it even induced coagulation. This must be due to its nonspecific interaction with blood proteins. Unlike ONs with single-point thio-modifications, fully-modified thiophosphoryl ONs (GQ-forming thio-ONs in particular) exhibit strong non-specific binding to proteins (to heparin-binding proteins in particular) [16]. To further investigate the impacts of full and partial thio modifications on aptamer properties, we performed thrombin-aptamer binding assays and molecular modeling of the complexes.

Binding with thrombin was studied by using a Photonic Crystal Surface Wave (PC SW) -based biosensor (http://pcbiosensors.com) with independent registration of the angle of total internal reflection TIR) from the liquid. Registration of a bounded optical wave propagating along a bioactive surface is a popular method for real-time label-free biomolecular binding detection [19]. PC SW based biosensors represent a promising alternative to the well-known surface plasmon resonance (SPR) based and optical waveguide based biosensors. Unlike SPR and optical waveguide techniques, PC SW on one dimensional photonic crystal (1D PC) technique lacks such disadvantages as sensitivity to analyte refractive index variations, which can be caused by minor changes in liquid temperature and composition, and the limitations which a penetration depth of an optical bounded wave in water imposes on the minimal size of the molecules under study. PC SW biosensors allow the determination of the effective adlayer thickness with high precision (parts of angstrom) [20], [21]. Here, we present the first implementation of this technique in DNA aptamer studies.

**Figure 2:**
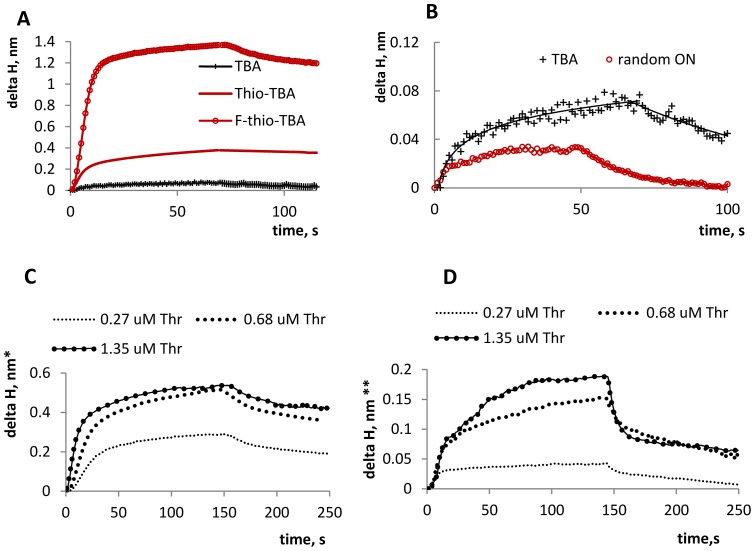
Evaluation of thrombin/aptamer interactions using PC SW biosensors. Delta H  =  increment of the effective adlayer thickness. A: Sensorgrams obtained upon aptamer binding with surface-immobilized thrombin. Delta H is normalized to thrombin adlayer effective thickness of 1 nm. B: Comparison of specific and nonspecific binding. Random ON is a G-rich ON of about the same length as TBA, which does not adopt monomolecular G-quadruplex structure under the specified conditions, as was proven by CD and UV-melting studies (Random ON  =  GGGAGGCTGATTCAGG). C: Sensorgrams obtained upon thrombin binding with surface-immobilized biotinylated TBA. Thr  =  thrombin. Delta H is normalized to the effective aptamer adlayer thickness of 0.25 nm. D: Sensorgrams obtained upon thrombin binding with surface-immobilized biotinylated thio-TBA. Delta H is normalized to the aptamer adlayer thickness of 0.5 nm. All experiments were performed in duplicate. Saturation level deviation did not exceed 5%.

Thrombin was immobilized on the working sensor surface. The surface was blocked and the solutions of the aptamers (all at the same concentration) were added. The obtained sensorgrams are shown in Figure 2 A, B. Despite the opposite biological effects (anticoagulation versus coagulation), both thio- and f-thio TBA bound with thrombin. However, their binding kinetics parameters, as well as the specific/nonspecific contributions to binding, appear to differ significantly. For the comparison of K_D_ values of thio-TBA/thrombin and f-thioTBA/thrombin complexes, an additional set of experiments was performed (Figure 2C, D). Biotynilated aptamers were immobilized on the streptavidin-coated biosensor working surface and different concentrations of thrombin were added. Aptamer fixation and the usage of thrombin solution as a mobile phase allowed us to achieve higher method sensitivity than the vice-versa scheme applied in our first set of PC SW experiments. Thio-TBA exhibits weaker affinity to thrombin than TBA (K_D_^TBA^ ≈250 nM±150 nM, K_D_^thio-TBA^ ≈900 nM±250 nM. For K_D_ determination see Figure S2. The obtained K_D_^TBA^ value is higher than that determined previously by solution methods [12], [22], presumably due to the partial shielding of the aptamer binding sites upon aptamer immobilization on the biosensor surface).

To clarify whether conformational or/and hydrophobicity changes underlie the effect of the thio-modification on thrombin-aptamer interaction, we performed molecular dynamics simulations for the thrombin/thio-TBA complex and evaluated the thrombin-aptamer binding energy. The initial models of the thrombin/thio-TBA complex were constructed using the high-resolution NMR-based structure of the unmodified complex [PDB: 1hao], in which TBA is bound to the protein via the TT-loops [23]. Although the inverted pattern of TBA interaction with thrombin has been reported [3], and the stoichiometry of the complex has been questioned [24], the most recent data reaffirm the TT-loops as the major recognition element in the 1:1 TBA-thrombin complex [5], [25]. To obtain structures of the modified complex, we substituted the oxygen atoms of the phosphate internucleotide linkages in the published structure with sulfur. Since thio-ONs exist as diastereomers, four structures were built for thio-TBA: RR, SS, RS and SR (R and S are the P configurations in the T3-T4 and the T12-T13 phosphorothioate internucleotide linkages).

**Figure 3:**
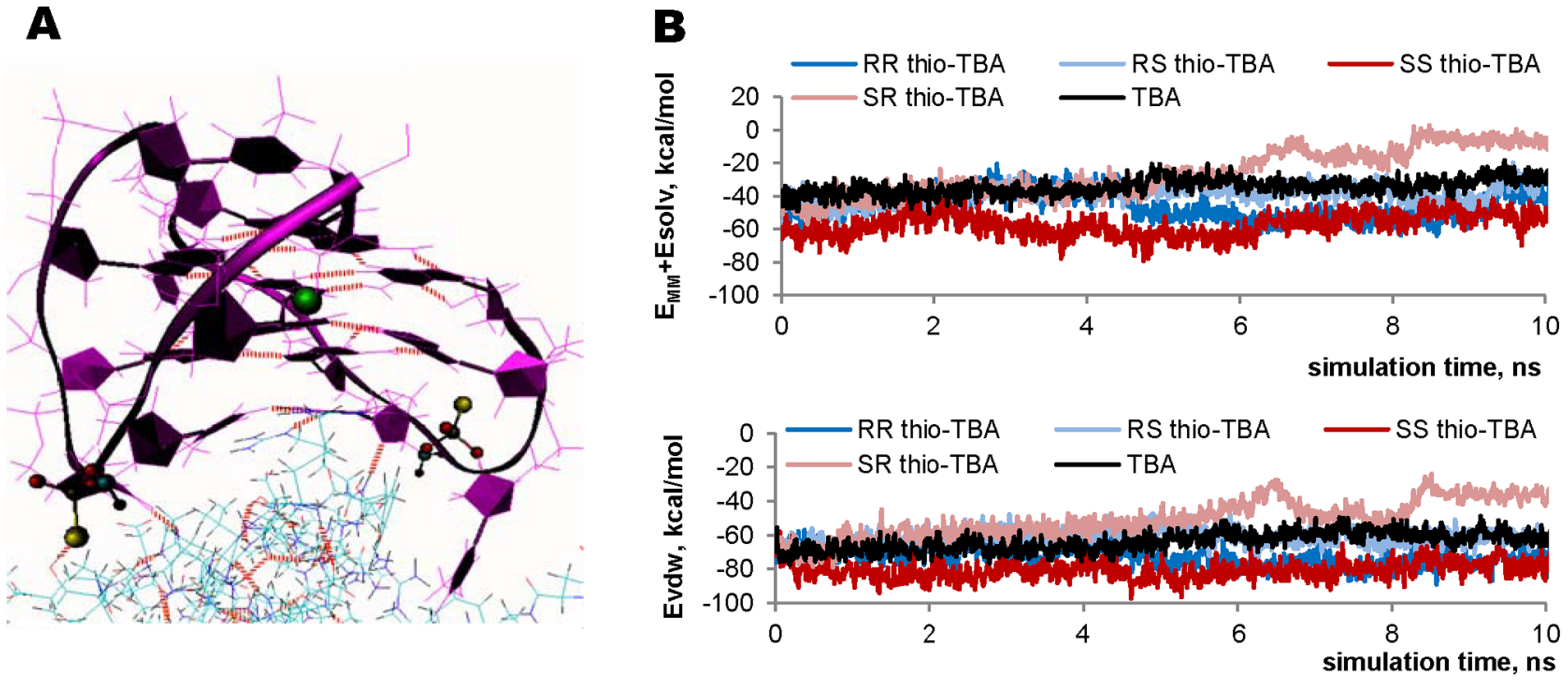
Thio-TBA/thrombin MD simulation results. A: The snapshot taken at 10 ns of RR thio-TBA/thrombin dynamics simulation. The aptamer does not dissociate from the protein, and no significant distortions in GQ structure can be seen. Red dotted lines are H-bonds. B: Plots of the thrombin-aptamer binding energy: the total binding energy (top) and the van-der-Waals contribution (bottom).

MD simulation results are summarized in Figure 3. Local charge rearrangement in the internucleotide fragment caused only slight changes in the electrostatic interactions in the binding site. The most significant difference between thio-TBA and TBA can be attributed to increased hydrophobicity of the sulfur atom. Figure 3A displays the fragment of the thrombin complex with RR thio-TBA. (RR is the only thio-TBA diastereomer, in which the sulfur atom participates explicitly in H-bonding with the Tyr114 OH group of thrombin). Thrombin-aptamer binding energies were calculated for all four diastereomers and compared with that of TBA. The electrostatic, van-der-Waals and solvation contributions to the binding energy were estimated. As evident from Figure 3B, diastereomers RR and SS seem to bind with thrombin more efficiently than TBA, primarily due to van-der-Waals interactions. However, the difference in the binding energies is relatively low. Thus, partial thio-modification does not cause any profound conformational changes in the aptamer and has little effect on its specific binding with the target protein. Nonspecific binding cannot be analyzed based on our MD simulation data, but it is likely that the hydrophobicity of the thiophosphoryl linkages plays an important role in those interactions.

To sum up, although they increase the ON life span in biological systems, chemical modifications were shown to decrease aptamer thermostability (Table 1) and specificity in most cases. While total internucleotide modification (f-thio-TBA) resulted in a complete loss of specificity, local modifications (thio-TBA, triazole-TBA and alpha-TBA) had moderate effects on bioactivity. These findings support the idea that modifications should be introduced locally. Extensive hydrophobic thiophosphoryl modification may have adverse effects and should be avoided.

### Addition of a duplex to the aptamer core structure

Duplex flanks can be added to various DNA secondary structures for their stabilization and, most important, for modeling their in-vivo surroundings [26]. (GQs in genomes are dynamic structures and exist within B-DNA. The model we describe in this paper (duplex-flanked GQ) lacks the GQ-opposing I-motif. Recent studies suggest that GQs and I-motifs may be mutually exclusive in-vivo [26] or mutually shifted in minisatellites. Thus, our GQ with duplex flanks is a somewhat simplistic model of native noncanonically structured DNA fragments, but in comparison with isolated single-stranded GQs it is relatively close to in-vivo state).

**Figure 4:**
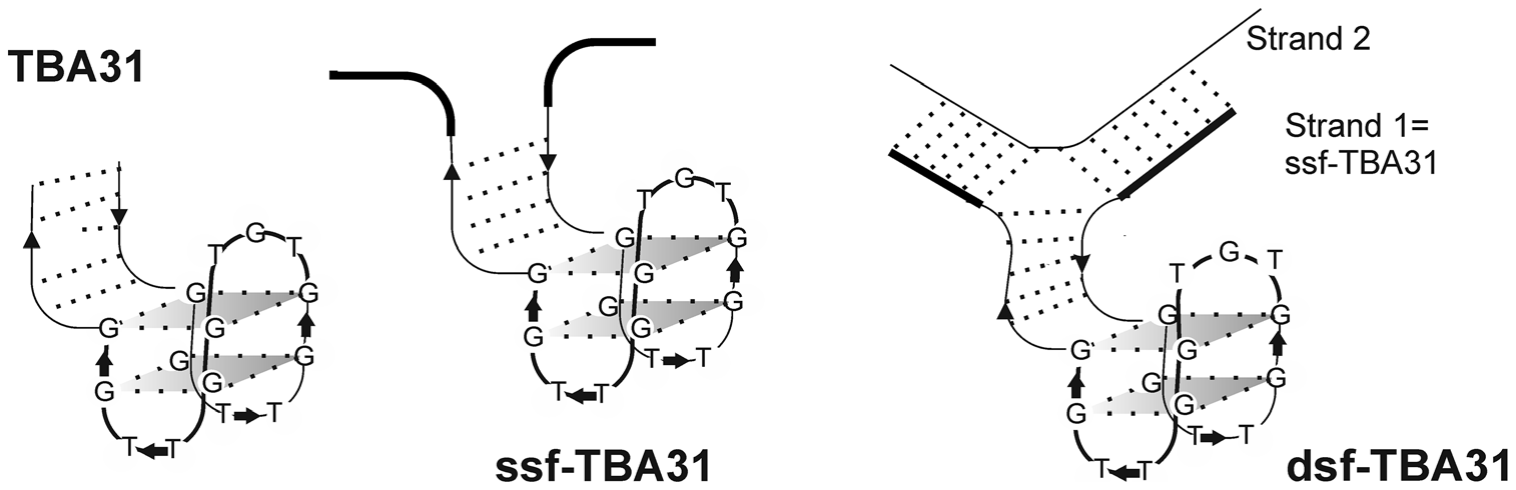
Schematic representation of TBA analogs with a duplex module and flanks.

Flanks are known to influence thermostability of noncanonical DNA structures and their affinity to proteins [27], [28]. While single-stranded flanks generally tend to destabilize GQs [27], duplexes may enhance GQ stability. One clear example of a stabilized aptamer is TBA31 (Figure 4, Table 2) [28], the monomolecular TBA analog bearing a duplex module adjoined to the quadruplex core (T_m_ = 56°C, see supporting information for the melting curve). Here, we designed the TBA31 analog, dsf-TBA31, with double-stranded flanks on both sides. Dsf-TBA31 is a bimolecular structure (Figure 4, Table 2). To obtain correctly folded dsf-TBA31, we first annealed 31TBA with single-stranded flanks (ssf-TBA31) under monomolecular-GQ-favoring conditions and then mixed it with the second strand at a ratio of 1:1. The flanking sequences did not contain any TBA-complementary fragments and could not interfere in the TBA-module folding.

**Table 2:** Sequences, MALD-TOF MS data and thrombin-time values of TBA analogs with a duplex module and flanks.

aptamer	sequence, 5’–3’*	m/z, found (calculated for [M + H]^+^)	thrombin time, s
31TBA	**CACTGGTAGGTTGG TGTGGTTGGGGCCAGTG**	9710 (9711)	62±2
ssf-TBA31	TCACCTGCACGCCAAGTGTG **CACTGGTAGGTTGGTGTGGTTGGGGCCAG TG**GCCAAGTGTG	18984(18981)	30±1
dsf-TBA31	Strand 1 = ssf-TBA31, Strand 2: *TGCAGGTGAC*CACACTTGGCCACA CTTGGCGTGCAGGTGA*ACACTTGGCG*	strand2:15430 (15429)	62±1
NegContr	Strand1:TCACCTGCACGC CAAGTGTGCGCCAAGTGTG; Strand 2 = that of dsf-TBA31	strand1:9498 (9498)	11±1**

* The TBA31 fragment is in bold. Overhanging single-stranded fragments of dsf-TBA31 Strand 2 are in Italics. The overhangs (sticky ends) were introduced in dsf-TBA31 structure to open up the possibility for the assembly of polyvalent supramolecular structures.

** the same as without any ON.

**Figure 5:**
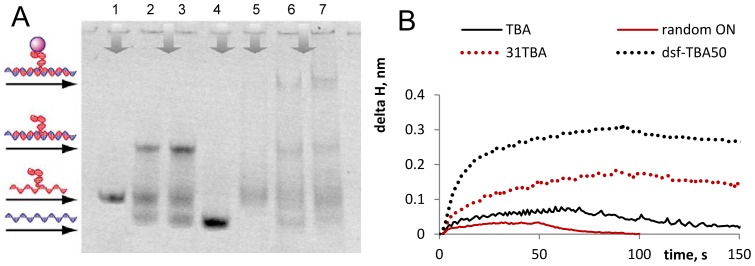
The assembly of dsf-TBA31 and its binding with thrombin. A: EMSA results illustrating the formation of the intermolecular dsf-TBA31 structure and its complex with thrombin. 1– ssf-TBA31; 2 and 3– dsf-TBA-31 (ssf-TBA-31+ strand 2); 4– strand 2 of dsf-TBA31; 5– ssf-TBA31+ thrombin; 6 and 7– dsf-TBA31+ thrombin. B: PCSW-sensorgrams illustrating dsf-TBA31 binding with thrombin. The sensorgrams were obtained upon aptamer interaction with surface-immobilized protein. Random ON  =  GGGAGGCTGATTCAGG. Delta H  =  increment of the effective adlayer thickness.

The assembly of dsf-TBA31 and its binding with thrombin were demonstrated in a band-shift assay (Figure 5A). As evident from the figure, the yield of the double-stranded structure does not exceed 50–60% under the conditions of electrophoresis. Dsf-TBA31 complex with thrombin is clearly visible in the electropherogram, while ssf-TBA31 appears to have only weak, if any, affinity to thrombin. The intermolecular structure of dsf-TBA31 was additionally confirmed by AFM (Figure S3), and the binding with thrombin was confirmed using PC SW biosensors (Figure 5B).

Anticoagulant activities of the double-module aptamers were evaluated by thrombin-time tests (Table 2). TBA31 was a more efficient thrombin inhibitor than TBA15, which agrees with the data in the literature [28]. The activity of dsf-TBA31 was two-fold higher than that of ssf-TBA31 and equal to that of unmodified TBA31. These results allow us to conclude that the addition of the duplex module and duplex flanks to the core aptamer structure does not impede its binding with the target protein, while single-stranded flanks are disadvantageous.

## Discussion

Two approaches for optimizing DNA aptamers – chemical modification and the addition of a duplex module and flanks – were compared. The first approach was illustrated by thiophosphoryl, triazole and ‘anomeric’ modifications of TBA. The first two modifications have been described before by our group [11], [12]. However, physicochemical properties and inhibitory activities of the three types of modified aptamers were examined under the same conditions for the first time. This enabled us to perform the comparative analysis, which provided deeper understanding of the advantages and disadvantages of the approach. The major advantage is improved biostability of chemically modified aptamers. The disadvantages include poor predictability of the modification effects and the need for identifying preferential positions of modification individually for each particular aptamer. (In the case of TBA, GQ loops, especially the central one, appear to be the preferential positions.) Additionally, chemical modifications (at least the three types discussed) are rarely beneficial in terms of aptamer thermostability and selectivity. All TBA analogs apart from thio-TBA demonstrated slightly decreased Tm values compared to the unmodified aptamer (Table 1). Selectivity loss is well-known for thio-ONs, and it is the most likely reason for the reversed activity of f-thio-TBA. In conclusion, while extensive studies of a larger number of modifications may provide a more balanced view on the ‘chemical’ approach, our findings suggest that that it is generally promising in the case of well-explored DNA ligands. However, the approach has its limitations and drawbacks. In model studies or in the case of poorly characterized aptamers, its application may be challenging.

The implementation of the second approach is less dependent on the specific features of a particular aptamer. A duplex module can theoretically be added to any DNA secondary structure. This approach was illustrated by the known monomolecular (TBA31) and the new bimolecular (dsf-TBA31) TBA analogs with duplex fragments. The most important advantage of the ‘duplex module’ approach is relative similarity to in-vivo state (GQs with duplex flanks). The approach seems to have greater potential in fundamental studies of DNA conformation and DNA-protein interactions than in drug design.

It should be mentioned that the ways of aptamer optimization are not limited to the two approaches discussed here. A number of original strategies, such as aptamer fixation on nanoparticles and arrays [29], have been reported recently. However they mainly address aptamer handling and delivery rather than stability or target affinity. Aptamer oligomerization [30] seems to be a promising strategy for improving affinity, but increased avidity may result in increased toxicity and immunogenity. The relative characteristics of the two approaches described in this study are summarized in Table 3.

**Table 3:** Comparison of the two approaches to DNA ligand optimization.

aptamer characteristics	approach 1 (chemical modification)	approach 2 (addition of a duplex)
enhanced thermostability	±	+
resistance to biodegradation	+	not analyzed
enhanced affinity to target protein	±	−
resemblance to *in-vivo* state	−	+

## Conclusion

Two approaches for improving the stability and the target affinity of DNA ligands were illustrated by the optimization of the thrombin binding aptamer. The commonly applied approach (chemical modification) appeared rather efficient. The three types of modifications, which ensure increased ON biostability according to literature data [11], [12], [18], were well-tolerated in terms of bioactivity. (The only exception was the thiophosphoryl modification throughout the chain, which resulted in a reversed biological effect of the aptamer.) However, application of this approach to less-known aptamers with poorly characterized mechanisms of action would be complicated. The relatively new approach (addition of a duplex module) is potentially applicable to different kinds of DNA ligands and is of significant interest for fundamental biochemical studies, particularly for modeling the behavior of GQs in duplex media.

## Materials and Methods

### ON synthesis, purification and MS analysis

All phosphodiester and thiophosphoryl ONs were synthesized as in [11]. Triazole-TBA was synthesized as in [12]. ‘Alpha’-TBA was synthesized on an Applied Biosystems 3400 DNA synthesizer (USA) following standard phosphoramidite protocols using standard commercial reagents and modified phosphoramidites. The alpha-thimidine phosphoramidite was purchased from ChemGenes. All oligonucleotides were purified by preparative scale reverse-phase HPLC on a 250 mm ×4.0 mm Hypersil C18 column with detection at 260 nm and a 12–24% gradient of CH_3_CN in 0.1 M ammonium acetate buffer. The dimethoxytrityl protection group was removed via treatment with 80% acetic acid (20 min), and the detritylated oligonucleotides were further purified in a 0–12% gradient of CH_3_CN in 0.1 M ammonium acetate buffer. The purity of all oligonucleotides was determined to be ≥95% by HPLC. The peak purity was confirmed by the UV spectra of the peak. The MALDI TOF mass spectra of the oligonucleotides were acquired on a Bruker Microflex mass spectrometer in linear mode (+20 kV). Each spectrum was accumulated using 200 laser shots (N_2_ gas laser, 337 nm). A solution of 35 g/ml of 3-hydroxypicolinic acid with dibasic ammonium citrate was used as the matrix.

### UV melting

The oligonucleotides were dissolved in a 20 mM sodium phosphate buffer containing 100 mM KCl (pH 7.5). The oligonucleotide single strand concentrations were calculated from the absorbance measured above 80°C and the extinction coefficients, which were approximated using the nearest-neighbor model. The samples were denatured at 95°C for 5 min and cooled quickly to 15°C prior to measurements. The UV melting curves were recorded on a Jasco V-550 spectrophotometer equipped with a thermostated cuvette holder. The absorbance was registered at λ = 295 nm every 0.5°C across the 15–90°C temperature range. The melting temperatures of the quadruplexes were defined by performing a fitting procedure using the two-state model for monomolecular melting [31] in KaleidaGraph version 4.0.

### TT assay

The thrombin time (TT) was measured using the Renam Thrombin-TEST assay kit, following the published procedure [12] and the Renam protocols. Citrate-stabilized plasma was obtained as specified in the ‘MST’ section. The plasma (100 µL) was incubated for 120 s at 37°C, followed by the addition of the aptamer to a final concentration of 0.1–3 μM and thrombin (6u). The clotting time was then measured using a Unimed MiniLab-701 coagulation analyzer.

### PC SW biosensors

The following multilayer stack were used as 1D photonic crystal for the detection of aptamer binding with thrombin: substrate/(LH)^3^L’/water, where L is a SiO_2_ layer with thickness d_1_ = 183.2 nm, H is a Ta_2_O_5_ layer with d_2_ = 111.2 nm and L’ is a final layer of SiO_2_ with d_3_ = 341.6 nm. This 1D PC structure was theoretically predicted by using impedance approach [32] and made by magnetron sputtering. The prism and the 1D PC substrate are made from BK-7 glass. PC SW excitation is induced by polarization-maintaining fiber-coupled diode laser at λ = 658 nm. The surface of the 1D PC slides was prepared by rinsing with water and subsequently Isopropyl alcohol, drying by compressed air and 10 minutes cleaning in Diener electronic Zepto plasma cleaner with 100W power under 0.8 mbar air pressure. Then the surface was functionalized by real-time immersion into and subsequent washing with water solutions of polyallylamine (0.1 mg/ml) and glutaraldehyde (0.1%). All reagents are commercially available in Sigma Aldrich.

#### PC SW experiments with immobilized thrombin

Thrombin was immobilized on the modified 1D PC surface as follows. Thrombin solution (50 ug/mL) in a working buffer (10 mM NaHPO_4_ (pH 7.4), 140 mM NaCl, 3 mM KCl) was injected and pumped through the working chamber until binding signal saturation. The surface was blocked afterwards by BSA solution (50 ug/mL) and the chamber was rinsed with the working buffer. The aptamer solution (10 uM) in the buffer was injected, pumped through the chamber for 1 minute at a flow rate of 1,5 uL/sec and the chamber was rinsed with the buffer for 1 more min (flow rate 1,5 uL/sec).

#### PC SW experiments with immobilized aptamer

Streptavidin was immobilized on the modified 1D PC surface using the method described for thrombin in the above subsection and the surface was blocked by BSA solution in the working buffer. Aptamer solution (10 uM) in the buffer was pumped through the working chamber until the signal saturation. The surface was washed with the buffer and blocked with Random ON solution (Random ON  =  GGGAGGCTGATTCAGG). The thrombin solution in the buffer was injected afterwards. Different concentrations of thrombin (10, 25 and 50 ug/mL) were used. Thrombin solution was ran over the aptamer-coated 1D PC surface for 150 s (time required for the signal saturation), then the chamber was washed with the buffer for additional 100 s (flow rate  = 1,5 uL/min).

### Molecular modeling

Partial atomic charges of the thiophosphoryl linkage atoms were obtained by single-point energy quantum mechanics calculations. Electron density in thiophosphoryl linkages (including C3’ and C5’) was calculated by ab initio quantum mechanical methods (density functional theory (DFT) with hybrid exchange-correlation functional B3LYP [33], [34], [35] and Hartree-Fock (HF) theory [36]). Basis sets 6-31G(d) and 6-311G(d) were used in DFT/B3LYP and HF calculations. To compute partial atomic charges based on the calculated electron density distribution, three calculation schemes were applied: Mulliken’s population analysis scheme (MPA) [37], Natural population analysis scheme (NPA) [38], and CHELPG (Charges from Electrostatic Potentials using a Grid based method) [39]. We analyzed various combinations of the above methods and calculation schemes taking into account that 1) calculated partial charges of non-modified nucleoside fragments are supposed to be close to standard AMBER values and 2) diastereomers are supposed to have different partial charges on thiophosphoryl O- and S atoms and slightly different charges on thiophosphoryl P. The analysis showed that B3LYP/6-31G(d) in combination with MPA is the most adequate approach to calculating atomic charges in our case, and it was finally used in this study. All quantum mechanics simulations were carried out using the Gaussian 09 program [40]. Since the calculated charges on atoms other than O-, P and S were very close to standard AMBER values, the standard values were used.

The molecular dynamics simulations (MD) were performed with the Amber 8 suite with ff99SB and parmbsc0 force fields as described in [12]. The trajectory length was 8 ns. Snapshot visualization and hydrogen bond analysis were performed using VMD [http://www.ks.uiuc.edu/Research/vmd/] with a donor-acceptor distance of 3 Å and an angle cutoff of 20 degrees. Snapshots were taken every 0.1 ns.

#### MM-GBSA analysis

MM-GBSA method was used to calculate the thrombin/TBA and thrombin/thio-TBA binding free energies. In this approach, binding free energy of a complex is calculated according to the formula in Eq.1.
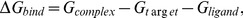
(1)where *G_complex_, G_target_* and *G_ligand_* are the energies of the complex (thrombin/aptamer), target (thrombin) and ligand (aptamer) respectively. Each term in Eq.1 can be represented as shown in Eq.2.
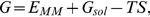
(2)where *E_MM_*, *G_sol_* and *TS* are the total mechanical energy of the molecule in gas phase, the free energy of hydration and the entropic contribution respectively. *E_MM_* was calculated as the sum of electrostatic energies, van der Waals energies and the energies of internal strain (bonds, angles and dihedrals) by using the molecular-mechanics approach. *G_sol_* was calculated as the sum of polar (*G_polar_*) and nonpolar (*G_nonpolar_*) terms. The electrostatic contribution to the hydration energy Gpolar was computed by the Generalized Born (GB) method [40] using the algorithm developed by Onufriev et. al. [41], [42] for calculating effective Born radii. The non-polar component of the hydration energy Gnonpolar, which includes solute-solvent van der Waals interactions and the free energy of cavity formation in solvent, was calculated by using the following formula: 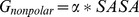, where SASA is the solvent accessible surface area. SASA was computed by the LCPO method [43] with α = 0.00542 kcal/mol^−1^ Å^−2^. Since the replacement of only two oxygen atoms with sulphur is unlikely to cause significant changes in the internal strain energy and the entropic component, these two terms were omitted in our study. Snapshots taken from a single trajectory of the complex MD simulation were used for the calculations of the binding free energy. Dielectric constants for gas-phase and water-phase calculations were set to 1 and 80 respectively.

### Dsf-TBA31 assembly and EMSA

To obtain correctly folded dsf-TBA31, we annealed ssf-TBA31 under monomolecular-GQ-favoring conditions (heated it to 95°C and cooled quickly to room temperature) in 25 mM Tris-HCl buffer (pH 8) containing 100 mM KCl, then added equimolar amount of the second strand and stored the solution at +4°C for 24 hours. To obtain aptamer-protein complexes, we incubated ssf-TBA31or dsf-TBA31 with 10 equivalents of human alpha-thrombin (Sigma-Aldrich) for 15 min at room temperature in a 25 mM Tris-HCl buffer (pH 8) containing 100 mM KCl, 5 mM MgCl_2_ and 2 mM beta-mercaptoethanol. The band shifts were resolved on a non-denaturing 8% polyacrylamide (19:1) gel in a Tris-HCl buffer (25 mM Tris·HCl, 10 mM KCl, 1 mM EDTA, pH 8.9). The gel was stained by SybrGreenII and analyzed using a GelDoc scanner (BioRad).

### Atomic force microscopy

The sample analysis by atomic force microscopy (AFM) was carried out at ambient conditions. Highly oriented pyrolytic graphite (HOPG) (10×10 mm^2^) freshly cleaved with adhesive tape prior to each experiment. HOPG surface was treated with 20 mkl of 0.01 mg/ml graphite modifier «GM» [44]. (Nanotyuning, Russia) and kept in a moist chamber for 10 minutes, then GM was removed in a stream of compressed argon. The adsorption of the ONs was carried out from a drop of the ON solution (20 mkl), which was applied to the surface of the substrate and kept for 1 minute. Then the ON solution was removed and the substrate was dried in the stream of compressed argon. The ON solution was prepared as follows: dsf-TBA31 (2 uM) was annealed in 20 mM Tris-HCl (pH 7) in the presence of 100 mM KCl and diluted 100 fold prior to loading onto HOPG. Low ON concentration was used to ensure separation of individual dsf-TBA31 molecules on the graphite. Scanning was performed on an Itegra Prima atomic-force microscope (NT-MDT, Russia) in the semi-resonant mode (resonant frequency 190–325 kHz) using high resolution silicon cantilevers (Nanotyuning, Russia) with the radius of the tip curvature about 1 nm and the angle at the top of the tip less than 22°. Free amplitude of the canteliver was in the range of 1–10 nm. AFM images were taken with 512 samples/line in 512 lines and scan rates of 0.5–1.0 lines/s. Signal processing and imaging was performed using NOVA 1.1 software (NT-MDT, Russia). The heights of the visualized objects were measured using the Image Analysis 2.0 module. When necessary, the AFM images were processed by flattening in order to remove the background slope and the contrast and brightness were adjusted.

## Supporting Information

Figure S1 **Physico-chemical characteristics of TBA and its analogs.** A: Melting curves of the aptamers. B: CD spectra of the aptamers. Δε is given per mole of nucleotides.
(PDF)

Figure S2 **Evaluation of KD for thrombin complexes with TBA (left) and thio-TBA (right).** Delta H is the equilibrium increment of the effective thrombin adlayer thickness. The steady-state affinity model was used to calculate KD values. Binding response at steady state (delta H at saturation in Figure 2) was plotted as a function of concentration and fitted with affinity isoterm model for 1:1 binding: deltaH  =  deltaHmax*C/(A+C), where C is equilibrium solution concentration of thrombin, A  = 1/KL (KL is Langmuir adsorption constant) and deltaHmax is binding signal at saturation. The fitting procedure was performed in Origin v8. Concentration at 50% saturation is KD.
(PDF)

Figure S3 **AFM-images of TBA with duplex flanks.** Left: negative control (dsf-TBA31 with a defective, i.e. lacking the quadruplex module, first strand). It appears as ∼1.3–2 nm sticks. A and B represent double-stranded and single-stranded ONs respectively. Right: dsf-TBA31. C is the correctly folded double-strand structure (∼1.5–2 nm-high bended stick with a nodule at the bending point). D is ssf-TBA31 =  strand 1 of dsf-TBA31 (∼1 nm-high curved stick with a nodule). The nodules are G-quadruplexes. The yield of the correctly-folded double-stranded dsf-TBA structures was very low under AFM conditions because of the low salt concentration used (AFM is incompatible with high salt concentrations).
(PDF)
