# Supplementary figures and images for: Comparison of the ‘Chemical’ and ‘Structural’ Approaches to the Optimization of the Thrombin-Binding Aptamer
Source: PLoS One. 2014 Feb 20;9(2):e89383. doi: 10.1371/journal.pone.0089383 (PMC3930721; doi:10.1371/journal.pone.0089383)

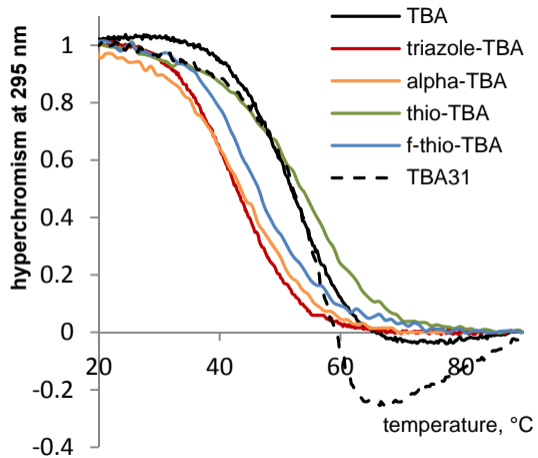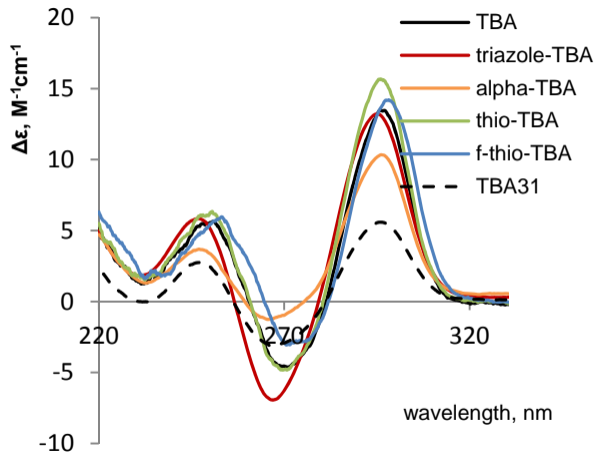

Supplement: Figure S1 — Physico-chemical characteristics of TBA and its analogs. A: Melting curves of the aptamers. B: CD spectra of the aptamers. Δε is given per mole of nucleotides. (PDF) [file pone.0089383.s001.pdf]

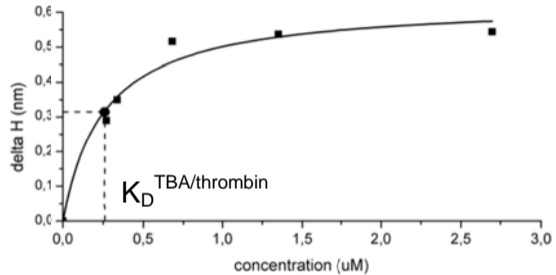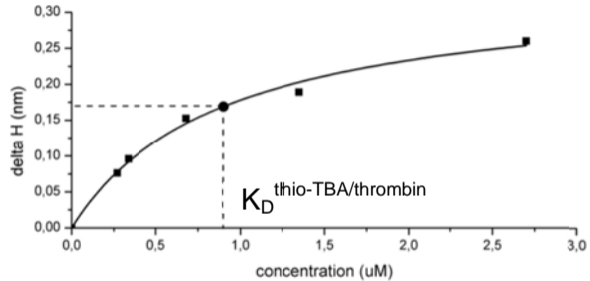

Supplement: Figure S2 — Evaluation of KD for thrombin complexes with TBA (left) and thio-TBA (right). Delta H is the equilibrium increment of the effective thrombin adlayer thickness. The steady-state affinity model was used to calculate KD values. Binding response at steady state (delta H at saturation in Figure 2) was plotted as a function of concentration and fitted with affinity isoterm model for 1:1 binding: deltaH = deltaHmax*C/(A+C), where C is equilibrium solution concentration of thrombin, A = 1/KL (KL is Langmuir adsorption constant) and deltaHmax is binding signal at saturation. The fitting procedure was performed in Origin v8. Concentration at 50% saturation is KD. (PDF) [file pone.0089383.s002.pdf]

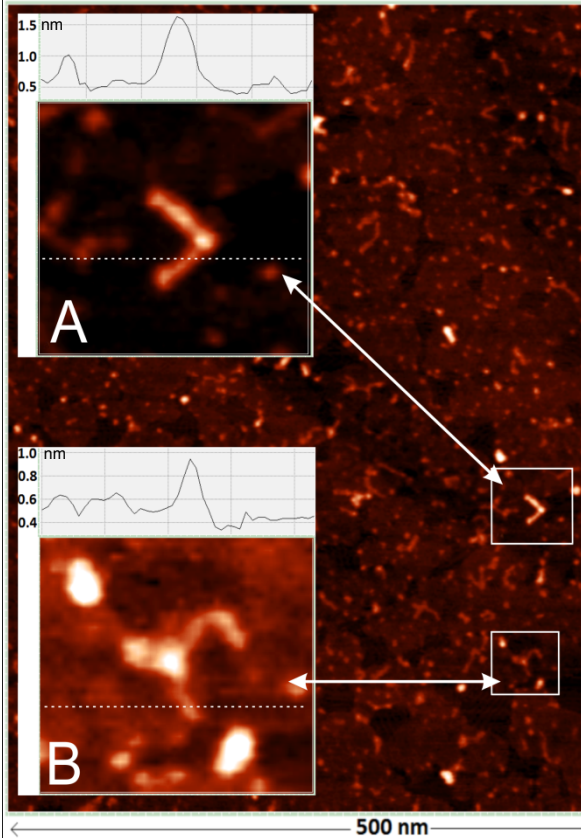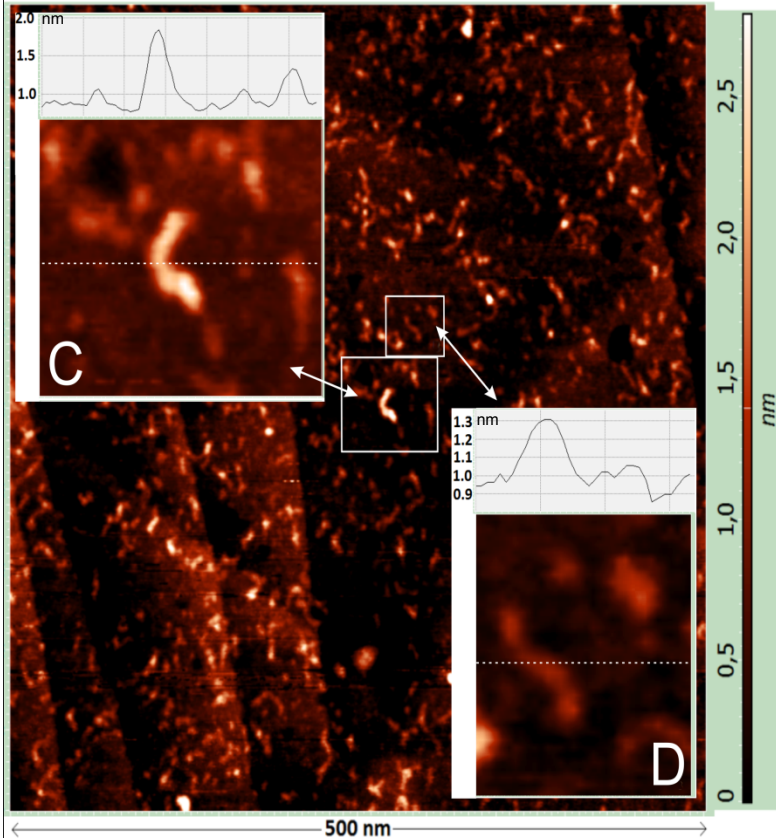

Supplement: Figure S3 — AFM-images of TBA with duplex flanks. Left: negative control (dsf-TBA31 with a defective, i.e. lacking the quadruplex module, first strand). It appears as ∼1.3–2 nm sticks. A and B represent double-stranded and single-stranded ONs respectively. Right: dsf-TBA31. C is the correctly folded double-strand structure (∼1.5–2 nm-high bended stick with a nodule at the bending point). D is ssf-TBA31 = strand 1 of dsf-TBA31 (∼1 nm-high curved stick with a nodule). The nodules are G-quadruplexes. The yield of the correctly-folded double-stranded dsf-TBA structures was very low under AFM conditions because of the low salt concentration used (AFM is incompatible with high salt concentrations). (PDF) [file pone.0089383.s003.pdf]
